# Daily perceived stress and sexual health in couples with sexual interest/arousal disorder

**DOI:** 10.1016/j.ijchp.2025.100582

**Published:** 2025-05-13

**Authors:** A. Girouard, S. Bergeron, J.S. Huberman, N.O. Rosen

**Affiliations:** a Department of Psychology & Neuroscience, Dalhousie University, Halifax, NS, Canada; b Department of Psychology, Université de Montréal, Montréal, QC, Canada; c Department of Obstetrics & Gynaecology, Dalhousie University, Halifax, NS, Canada

**Keywords:** Perceived stress, Sexual health, Sexual satisfaction, Sexual desire, Sexual distress

## Abstract

**Objective:**

Sexual Interest/Arousal Disorder (SIAD) is a common and distressing sexual health concern that may add stress to couple’s daily lives and maintain the low sexual desire/arousal. While stress has been linked to lower sexual desire, sexual satisfaction, and higher sexual distress in cross-sectional studies with community couples, dyadic research at the intersection of stress and sexual health is scarce. We examined the daily associations between perceived stress and sexual health among couples coping with SIAD.

**Methods:**

Women and gender diverse individuals with SIAD, and their partners, (*N* = 229, *M*_age_ = 34.94) completed online validated measures of perceived stress, sexual desire, satisfaction, and distress for 56 days. Data were analyzed with residual dynamic structural equation modeling guided by the Actor-Partner Independence Model.

**Results:**

On days when individuals with SIAD perceived more stress than usual, they and their partners reported lower sexual satisfaction and desire, and higher sexual distress. On days when partners perceived more stress, they and individuals with SIAD reported lower sexual satisfaction and desire, and partners also reported higher sexual distress. Between-person results across the diary period showed similar patterns, though fewer effects overall.

**Conclusion:**

Findings highlight dyadic processes in sexual health whereby one partner’s daily stress was associated with lower sexual health for both partners. Stress might orient partners away from sensitive support provision and interfere with intimacy, which is crucial to sexual health. Interventions fostering sexual health in couples with SIAD should include a focus on stress management.

As defined by the World Health Organization (WHO, 2017), “sexual health is a state of physical, emotional, mental and social well-being about sexuality; it is not merely the absence of disease, dysfunction or infirmity”. A key dimension of overall health, sexual health is also an important determinant of quality of life (Flynn et al., 2016). One of the most relevant contexts to examine the unique psychological and physical benefits of sexual health (Diamond & Huebner, 2012) is within an intimate relationship, as individuals who are married report better health outcomes and quality of life than those who are unmarried (Holt-Lunstad et al., 2017; Kiecolt-Glaser & Wilson, 2017). However, having a partner with whom one is sexually active does not guarantee benefits or necessarily protect against harm, as interpersonal stressors in relationships are common and rated amongst the most noxious (Cohen et al., 2019). Sexual dysfunctions represent a major strain for couples that can threaten their sexual health and wellbeing (Rosen et al., 2019), and are one of the most common reasons why couples seek therapy (Emond et al., 2024). In turn, stress is related to lower sexual satisfaction (Bodenmann et al., 2010), sexual desire (Ferreira et al., 2015), and higher sexual distress (Kalmbach et al., 2019). Yet, despite interpersonal conceptualizations of sexual dysfunction (Rosen & Bergeron, 2019) and stress (Shrout, 2021), no previous study has examined the dyadic associations between perceived stress and sexual health using ecologically valid methods in a sample of couples coping with sexual dysfunction. In the current study, we examined the daily associations between perceived stress and three facets of sexual health—sexual satisfaction, desire and distress—in couples coping with clinically low sexual desire and/or arousal difficulties, i.e., Sexual Interest/Arousal Disorder^1^ (SIAD).

## Sexual health in couples coping with SIAD

SIAD is the most common sexual dysfunction, affecting up to 23% of cisgender women (Witting et al., 2008). Similar rates have been found among transgender women (Kerckhof et al., 2019; Wierckx et al., 2014), yet evidence is lacking for gender diverse individuals. As per the Diagnostic and Statistical Manual of Mental Disorders (DSM-5), SIAD is characterized by three or more of the following six symptoms, which indicate an absence or reduction of: 1) interest in sexual activity; 2) sexual thoughts or fantasies; 3) initiation of sexual activity and lack of responsivity to partner’s attempts; 4) pleasure or excitement in at least 75% of sexual encounters; 5) genital/non-genital sensations in at least 75% of sexual encounters; 6) arousal/interest in response to internal or external sexual cues (American Psychiatric Association, 2013). Compared to controls, individuals with SIAD report greater healthcare costs (Foley et al., 2010), depressive symptoms, anxiety, and lower relationship satisfaction (Parish & Hahn, 2016; Sarin et al., 2016). Yet, recent theoretical models such as the Interpersonal Emotion Regulation Model of Women’s Sexual Dysfunction (IERM; Rosen & Bergeron, 2019), highlight that both partners contribute to and are impacted by low sexual desire. Empirical findings support this dyadic conceptualization of SIAD, as both partners report lower sexual satisfaction (i.e., the subjective evaluation of one’s sexuality and its subsequent affective response; Pascoal et al., 2018) and sexual desire (i.e., the motivation to engage in sexual activity or experience sexual intimacy; Mark, 2014) as well as higher sexual distress (i.e., the negative emotions arising in the face of one’s sexual experiences; Derogatis et al., 2002) compared to unaffected couples (Rosen et al., 2019).

## Stress and sexual health

The transactional stress theory postulates that stress is a response to demands of one’s environment that have been evaluated as beyond one’s ability to cope (Lazarus & Folkman, 1984). Everyday stressors are inevitable, but their sexual health repercussions vary based on individual differences in subjective appraisal of the threat (Cohen et al., 2019; Kiecolt-Glaser et al., 2020). Stress contributes to poorer health outcomes (O’Connor et al., 2021) and has been identified as a crucial factor related to lower sexual satisfaction in cross-sectional and longitudinal studies (Abedi et al., 2015; Bodenmann et al., 2010), and to higher sexual distress in a cross-sectional study with post-menopausal women (Kalmbach et al., 2019). The evidence linking stress to sexual desire is more nuanced (see Mark & Lasslo, 2018 for review), with some studies reporting lowered desire (Ferreira et al., 2015), and another showing higher desire in men but lower desire in women when they report more stress (Raisanen et al., 2018). Yet, no studies have investigated the sex-stress link in the everyday life of individuals coping with a sexual dysfunction, which could inform valuable treatment options (Mües & Nater, 2020).

Couples are known to shape each other’s stress appraisal and reactivity (Kiecolt-Glaser et al., 2020) through either synchrony, transmission, or co(dys)regulation (Butler, 2015). While the support provided by a romantic partner can buffer against the negative effects of stress, partners can also pass on their stress to one another (Butler, 2015). Important frameworks including interdependence theory (Kelley & Thibaut, 1978) and the dyadic biobehavioral stress model (Shrout, 2021) guide research on the reciprocal influence of both partners’ stress on their individual and relational health. These models illustrate that both individual (e.g., life adversity) and dyadic factors (e.g., attachment, communication) inform romantic partners’ perceptions and reactions to stress, which then act on relationship outcomes (e.g., conflict or reassurance), with implications for both partners’ psychological, behavioral, and biological health (Shrout, 2021). In support of this model applied to sexual health, in 102 heterosexual couples, greater self-reported stress was associated cross-sectionally with one’s own and a partner’s lower sexual satisfaction (Karakose et al., 2023). Moreover, in mixed-sex/gender couples transitioning to parenthood, lower perceived stress was associated cross-sectionally with one’s own and a partner’s higher sexual satisfaction and desire, and fewer sexual concerns (Tavares et al., 2019). Lastly, everyday stress has been identified as a barrier to treatment adherence to interventions for low sexual desire (Meyers et al., 2022), and might therefore be a central element to a negative feedback loop propelling sexual health difficulties in an already burdened population – couples coping with SIAD.

Nearly all past studies examining the contribution of stress to sexual health used cross-sectional methodologies, whereas daily diary designs are more ecologically valid, minimize recall bias (Boynton & O’Hara, 2018), and consider day-to-day stress fluctuations (Shrout, 2021). Also, whilst we know that sexual dysfunctions are inherently relational (Rosen & Bergeron, 2019) and that stress is a shared experience in committed relationships (Shrout, 2021), most past studies neglected to include both partners or clinical samples, leaving a gap in the literature regarding dyadic studies of couples coping with sexual dysfunction.

## Stress and sexual health in couples coping with SIAD

SIAD represents a stressful reality considering its personal, relational and financial costs (Rosen et al., 2019). Consistent with the transactional model of stress (Lazarus & Folkman, 1984), sexual dysfunction may be a risk factor for worsened stress (Tavares et al., 2019). In addition, exposure to daily stressful life events is thought to exacerbate preexisting conditions such as sexual dysfunction (Cohen et al., 2019). In qualitative studies of women in heterosexual relationships, stress was amongst the most frequently stated factors that interfered with sexual desire (Ferreira et al., 2015; Goldhammer & McCabe, 2011). Another qualitative study with 31 lesbian, bisexual, and heterosexual women revealed a tipping point where stress shifted from being protective for desire to diminishing desire (Rosenkrantz & Mark, 2018). Also, a recent study that analyzed 1279 open-ended answers from women and gender diverse individuals with SIAD and their partners identified the link between stress and desire as an important research priority (Shimizu et al., 2024). Yet, as previous qualitative studies on stress and sexual desire focused on non-clinical samples of individuals, a significant gap exists regarding quantitative dyadic investigations of perceived stress and sexual health in SIAD.

## Current study

The current study examined the associations between daily variations in perceived stress and sexual satisfaction, sexual desire, and sexual distress among women and gender diverse individuals who met criteria for SIAD and their partners, across a 56-day diary period. We examined these associations within-person (i.e., variation within individuals/co-occurring variation over time), which capitalizes on the robust daily diary methodology, and between-person (i.e., variation across individuals/averaged over time). In line with prior research and theory (Bodenmann et al., 2010; Ferreira et al., 2015; Kalmbach et al., 2019), we predicted that on days when women and gender diverse individuals with SIAD, or their partners, reported higher levels of perceived stress compared to their own average (i.e., within-person level), both partners would report lower sexual satisfaction, lower sexual desire, and higher sexual distress that day. In addition, across the diary period (i.e., between-subject level), we expected that reporting higher levels of perceived stress would be linked to one’s own and a partners’ lower sexual satisfaction, lower sexual desire, and higher sexual distress.

## Method

The present study was part of a larger study examining psychosocial factors and the sexual and relational well-being of couples coping with SIAD in two North American cities [*masked for review*]. There is one publication to date that used the baseline data [*masked for review*], one that included both baseline and 6-month follow-up data [*masked for review*] and one that used the daily diary data [*masked for review*], none of which overlapped with the current research questions. The research protocol was the same across the two sites and all procedures were approved by both institutional review boards. This study was pre-registered on the Open Science Framework (OSF): https://osf.io/kwby6/?view_only=b99308f5b36a4acabb5fb312031393ac.

### Participants

A total of 292 North American couples were recruited between November 2020 and May 2022 through print and online sources. Inclusion criteria involved: being 18 years of age or older, speaking English and/or French fluently, having access to a personal e-mail account, and having a minimum of four weekly in-person contacts with one’s partner per month. Also, one member of the couple, either a woman or a gender diverse individual who was assigned female at birth, had to meet DSM-5 criteria for SIAD (American Psychiatric Association, 2013). Exclusion criteria included: ongoing treatment for sexual challenges or fertility treatment, actively trying to conceive, being pregnant, breastfeeding, within one-year postpartum, or self-reporting a severe and untreated mental or physical illness.

Among the 603 couples who were initially interested, 263 couples were eligible and completed the baseline survey. While the larger project (OSF link masked) also included questionnaires at baseline, 6 month- and 12 month-follow-up, the current study focused solely on the daily data. A final sample of 229 couples (See Table 1 for descriptives) contributed daily diary data as some couples declined the invitation to the diary portion or were excluded due to insufficient data (see Fig. S1 for participant flowchart).

**Table 1:** Descriptive statistics (M ± SD or N [%]) of the sample’s (N = 229 couples) demographic variables.

	Women and gender diverse individuals with SIAD		Partners
Age (years)	34.52 ± 9.91	36.15 ± 10.49	
Gender			
Woman	221 (96.5%)	21 (9.2%)	
Man	1 (0.4%)	200 (87.3%)	
Indigenous (e.g., Two-Spirit)	2 (0.9%)	–	
Non-binary	10 (4.4%)	7 (3.1%)	
Additional†	2 (0.9%)	3 (1.3%)	
Sexual Orientation			
Asexual	4 (1.7%)	–	
Bisexual	28 (12.2%)	15 (6.6%)	
Heterosexual	159 (69.4%)	185 (80.8%)	
Lesbian/Gay	9 (3.9%)	16 (7.0%)	
Pansexual	14 (6.1%)	5 (2.2%)	
Queer	10 (4.4%)	5 (2.5%)	
Questioning	3 (1.3%)	3 (1.3%)	
Additional†	2 (0.9%)	–	
Culture			
African/Black	5 (2.2%)	8 (3.5%)	
American	5 (2.2%)	4 (1.7%)	
Asian	9 (3.9%)	14 (6.0%)	
Biracial/Multiracial	5 (2.2%)	6 (2.6%)	
English Canadian	96 (41.9%)	95 (41.5%)	
European	26 (11.3%)	25 (10.9%)	
Hispanic/Latin American	4 (1.7%)	8 (3.35%)	
Indigenous	5 (2.2%)	4 (1.7%)	
Québécois/French Canadian	107 (46.7%)	98 (42.8%)	
White	62 (27.1%)	72 (31.4%)	
Additional cultures‡	6 (2.6%)	5 (2.2%)	
Education (years from 1st grade)	16.10 ± 3.01	15.01 ± 3.19	
Length of SIAD (years)	7.30 ± 8.05	–	
Relationship Status			
Cohabitating/Married	215 (93.9%)		
Not cohabiting	14 (6.1%)		
Relationship Length (years)	9.36 ± 7.93		
Combined Annual Income			
$0-$39,999	39 (16.7%)		
$40,000-$79,999	62 (27.2%)		
$80,000-$119,999	67 (29.4%)		
>$120,000	56 (26.7%)		

*Note*. Participants could select multiple genders, sexual orientations, and cultures, thus, percentages may not add up to 100%. Cells containing only one participant are reflected in the “additional” categories to protect confidentiality. †The additional option provided was an open-ended response. ‡Additional options provided for culture included: Australian, Native Hawaiian/Other Pacific Islander, and an open-ended response.

### Procedure

The initial eligibility of interested couples was assessed through a telephone structured screening call with a member of our research team. If a couple was deemed eligible following the initial screening call, and was interested in participating, then the member experiencing low desire was invited to independently complete a 30- to 45-minute clinical interview via Zoom or telephone. Prior to the clinical interview, the individual attending the interview completed a consent form via Qualtrics Research Suite for both the clinical interview and participation in the study. During the semi-structured clinical interview, a research team member trained in assessing sexual difficulties and under the supervision of the principal investigators for the larger project (clinical psychologists) confirmed that the individual’s symptoms were in line with SIAD, and, if so, the couple was invited to enroll in the study. Once enrolled, both members of the couple independently completed the baseline survey followed by 56 days of diaries.

During the diary period, participants received a link to their email each day at 5 pm. in their respective time zone, which expired at 4 a.m. the following day. Couple members were instructed to complete the survey independently from their partner and prior to going to sleep to capture their experiences that day. A reminder link was sent nightly at 9 pm. for individuals who had not yet completed the survey. Retention strategies adapted from Dillman's (2007) tailored method were used throughout the daily diary period and consisted of weekly check-ins via phone or email. On average participants completed 41.46 (74.0%) of the 56 daily diary surveys. The surveys required an estimated 8 to 15 min to complete. Compensation was pro-rated across the daily diary period, and each participant was eligible to receive up to $120 CDN, paid via e-transfer or their choice of gift card.

### Measures

#### Sociodemographics

Participants reported on their demographics in the baseline survey, including sex, gender, sexual orientation, relationship status and duration, income, and culture. The person with SIAD also reported on their duration of SIAD symptoms.

#### Daily perceived stress

Daily perceived stress was assessed using the 4-item Perceived Stress Scale (PSS) (Cohen & Williamson, 1988). The PSS is a widely used measure to evaluate stress (Lee, 2012). The four-item instrument shows marginally acceptable psychometric properties compared to the PSS-10 and PSS-14, yet its use is considered appropriate and feasible in situations where a short questionnaire is required, such as in daily diaries (Lee, 2012). The PSS-4 has been used in prior daily diary studies and shown associations with health-promoting (e.g., eating and exercising) behaviors (Li et al., 2020). The PSS-4 asks respondents to describe their experience of stressful situations during that day using a five-point Likert-type scale (0 = never to 4 = very often). Mean scores are calculated after reversing positive items’ scores. A higher score indicates greater daily stress. In our sample, the scale demonstrated marginally acceptable internal reliability for within-person but adequate reliability for between-person, with similar magnitudes to past studies (Lee, 2012) (SIAD individuals: Ω_within-person_ = 0.69; Ω_between-person_ = 0.89, Partners: Ω_within-person_ = 0.67; Ω_between-person_ = 0.83).

#### Sexual satisfaction

Sexual satisfaction was assessed daily using the 5-item Global Measure of Sexual Satisfaction (GMSEX; Lawrence & Byers, 1995), in consideration of their sexual relationship that day. The GMSEX items included 7-point bipolar scales (e.g., 1 = very unpleasant to 7 = very pleasant). Mean scores were computed, with higher scores indicating higher sexual satisfaction. The scale demonstrated strong internal reliability (SIAD individuals: Ω_within-person_ = 0.94; Ω_between-person_ = 0.97, Partners: Ω_within-person_ = 0.92; Ω_between-person_ = 0.97).

#### Sexual desire

Sexual desire was assessed daily with four items adapted from the Sexual Desire Inventory (SDI-II; Spector et al., 1996) reflecting the cognitive, affective, and behavioral aspects of desire, consistent with SIAD symptoms and used in prior research (Jodouin et al., 2021). The items were rated on a scale of 1 = Not at all to 7 = A lot. Mean scores were computed with higher scores indicating higher sexual desire that day. The scale demonstrated strong internal reliability across our sample (SIAD individuals: Ω_within-person_ = 0.90; Ω_between-person_ = 0.96, Partners: Ω_within-person_ = 0.85; Ω_between-person_ = 0.96).

#### Sexual distress

Sexual distress was measured daily with the Sexual Distress Scale-Short form, which has been validated in women and men (Santos-Iglesias et al., 2020). This five-item measure asked participants to rate how often they felt concerns about their sex life today using a scale of 0 = Never to 4 = Always. Total scores ranged from 0 to 12, with higher scores indicating higher sexual distress. The scale demonstrated adequate internal reliability (SIAD individuals: Ω_within-person_ = 0.87; Ω_between-person_ = 0.95, Partners: Ω_within-person_ = 0.73; Ω_between-person_ = 0.93).

### Data analyses

First, descriptive statistics and correlations were computed with IBM SPSS Statistics (Version 26). Then, the main analyses were conducted in M*Plus* Version 8 (Muthén & Muthén, 1998). Analyses were informed by the Actor-Partner Interdependence Model (Cook & Kenny, 2005). To assess the associations between both partners’ perceived stress levels and one’s own and one’s partner’s outcome variables (i.e., sexual satisfaction, desire, and distress) across the 56 days of daily diaries, we used residual dynamic structural equation modeling (RDSEM; Asparouhov et al., 2018). We modeled our couples as distinguishable dyads based on SIAD diagnosis (women and gender diverse participants with SIAD vs their partners). RDSEM combines concepts from multilevel modeling, structural equation modeling, and time-series analysis. It uses residuals to estimate within-person autoregressive and cross-lagged regressions to account for the autocorrelation in residual errors (Asparouhov et al., 2018; McNeish & Hamaker, 2020). The predictor and outcome variables were all included in one model and split into two levels: Level 1 describes within-person effects (i.e., data from measures assessed daily for each participant), and Level 2 encompasses the between-person differences (i.e., individual differences in individual variables across the diary period; McNeish & Hamaker, 2020).

The model employed a Bayesian estimator and 5000 iterations. The Bayes estimator is a full-information estimator in M*Plus* (Muthén & Muthén, 1998), which optimally uses all available data for modeling, and, thus, is unbiased by missingness (Asparouhov & Muthén, 2010; Wang & Wang, 2019). To account for within-person stability, daily outcomes were regressed on the outcomes of the previous day (Bolger & Laurenceau, 2013), and on time since beginning the daily diaries, to account for potential upward or downward trends in the outcome variables as a factor of time (as recommended by McNeish & Hamaker, 2020). Additionally, this analysis utilized robust latent mean centering to partition within- and between-subject variance, including among predictors, outcomes, and lagged predictors (Asparouhov & Muthén, 2019). With the use of latent mean centering, the within-person effects can be interpreted as person-mean centered, i.e., variation relative to one’s own average across the diary period. All syntax and output are available on the OSF page and the datafile can be requested.

## Results

Descriptive statistics for participants’ demographic information can be found in Table 1.

Descriptives for all variables in the daily analyses as well as between-person correlations for all variables are in Table S1 (see Supplemental Materials), separately for each partner.

**Fig. 1:**
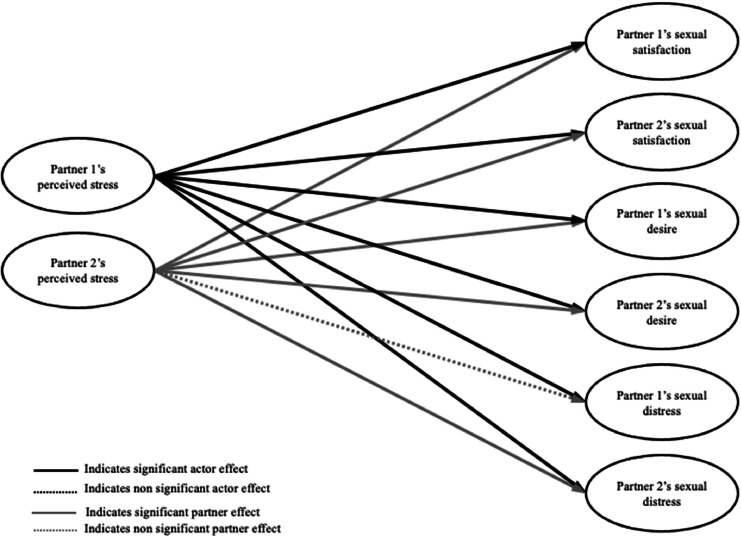
Diagram of daily within-level associations between perceived stress and sexual health outcomes.

*Note. Time was included as a control in the model, and correlations between all predictor variables and between all outcome variables were included in the model but omitted from the figure for the sake of visual clarity. Partner 1 refers to the individual with SIAD.

**Within-person level.** Controlling for yesterday’s outcomes, on days when women and gender diverse individuals with SIAD perceived more stress than usual (i.e., compared to their average levels across all diary days), they and their partners reported lower sexual satisfaction, lower sexual desire, and higher sexual distress. On days when partners perceived more stress than usual, both they and the individuals with SIAD reported lower sexual satisfaction and lower sexual desire than usual, and they themselves (but not the individuals with SIAD) reported higher sexual distress (see Table 2 and Fig. 1).

**Table 2:** Daily within-level and between-level associations between perceived stress and sexual satisfaction, sexual desire, and sexual distress (n = 229 couples).

Within-Level Associations	*B*	*SD*	95% *CI*
**Sexual Satisfaction**			
Actor effects			
Women and gender diverse individuals with SIAD	**-.26**	**.02**	**-.29, -.23**
Partners	**-.35**	**.02**	**-.39, -.31**
Partner effects			
Women and gender diverse individuals with SIAD	**-.14**	**.02**	**-.18, -.11**
Partners	**-.18**	**.02**	**-.21, -.14**
**Sexual Desire**			
Actor effects			
Women and gender diverse individuals with SIAD	**-.20**	**.02**	**-.24, -.17**
Partners	**-.28**	**.02**	**-.33, -.23**
Partner effects			
Women and gender diverse individuals with SIAD	**-.17**	**.02**	**-.21, -.12**
Partners	**-.16**	**.02**	**-.20, -.12**
**Sexual Distress**			
Actor effects			
Women and gender diverse individuals with SIAD	**.09**	**.01**	**.07, .12**
Partners	**.14**	**.01**	**.12, .16**
Partner effects			
Women and gender diverse individuals with SIAD	**.03**	**.01**	**.01, .05**
Partners	-.00	.01	-.03, .03

Between-Level Associations	*B*	*SD*	95% *CI*
**Sexual Satisfaction**			
Actor effects			
Women and gender diverse individuals with SIAD	-.18	.12	-.41, .06
Partners	**-.42**	**.14**	**-.69, -.15**
Partner effects			
Women and gender diverse individuals with SIAD	.27	.14	.00, .55
Partners	-.13	.12	-.36, .11
**Sexual Desire**			
Actor effects			
Women and gender diverse individuals with SIAD	-.06	.07	-.20, .07
Partners	-.13	.13	-.38, .13
Partner effects			
Women and gender diverse individuals with SIAD	**.34**	**.13**	**.08, .60**
Partners	.03	.07	-.11, .16
**Sexual Distress**			
Actor effects			
Women and gender diverse individuals with SIAD	**.36**	**.09**	**.17, .54**
Partners	**.43**	**.08**	**.27, .59**
Partner effects			
Women and gender diverse individuals with SIAD	.15	.08	-.01, .32
Partners	.09	.09	-.09 .27

*Note*. Significant effects (*p* < .05) are **bolded**. Actor effects refer to own perceived stress predicting own sexual health variables. Partner effects refer to own perceived stress predicting partner’s sexual health variables (i.e., the role noted in this table refers to the role of the perceived stress predictor variable). *B* = unstandardized betas; *SD* = standard deviation; *CI* = confidence interval

**Fig. 2:**
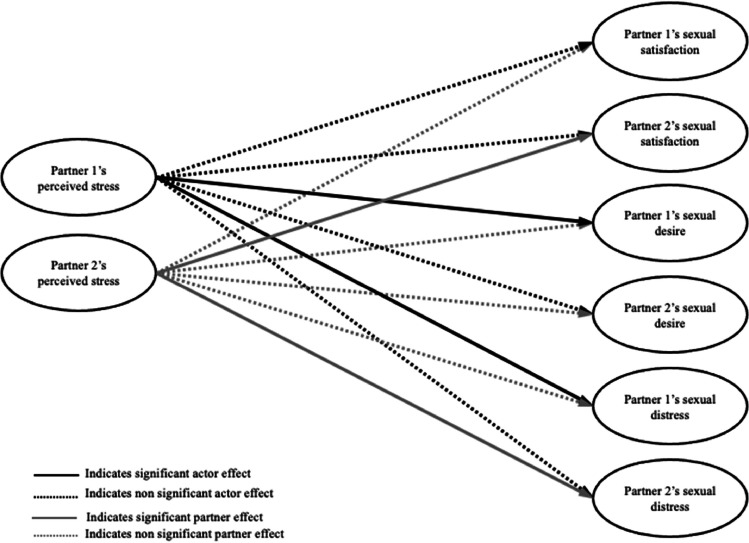
Diagram of daily between-level associations between perceived stress and sexual health outcomes.

*Note. Time was included as a control in the model, and correlations between all predictor variables and between all outcome variables were included in the model but omitted from the figure for the sake of visual clarity. Partner 1 refers to the individual with SIAD.

**Between-person level.** On average across the diary period, women and gender diverse individuals with SIAD who reported more perceived stress tended to report higher sexual distress themselves and their partners reported higher sexual desire. Individuals with SIAD’s perceived stress was not related to their own sexual satisfaction or desire, or to their partners’ sexual satisfaction or distress, across the diary period. Partners who reported more perceived stress, on average across the diary period, tended to report lower sexual satisfaction and higher sexual distress themselves, but their perceived stress was not related to their own sexual desire. Also, partners’ greater perceived stress was not related to the individual with SIAD’s sexual satisfaction, desire or distress (see Table 2 and Fig. 2).

## Discussion

In a sample of couples where one member met criteria for SIAD, this study examined whether daily perceived stress was related to three key aspects of one’s own as well as a partner’s sexual health—sexual satisfaction, sexual desire, and sexual distress. Controlling for the previous day’s outcomes, we found that on days when women or gender diverse individuals with SIAD perceived more stress than usual, they and their partners demonstrated lower indicators of sexual health on all domains compared to usual. Also, on days when partners perceived more stress than usual, both they and the individuals with SIAD reported lower sexual desire and lower sexual satisfaction, but only the partners (not the individuals with SIAD) reported higher sexual distress. When measures were averaged across the 56 days, we found fewer effects overall but similar patterns between perceived stress and sexual satisfaction and distress. Unexpectedly, however, individuals with SIAD’s higher perceived stress was associated with their partners’ higher desire. Results are in line with the Dyadic Biobehavioral Stress Model (Shrout, 2021) and prior research documenting detrimental effects of stress on sexual health (Karakose et al., 2023; Mark & Lasslo, 2018), while providing novel support for the dyadic interplay between stress and sexual health on a day-to-day level in the context of sexual dysfunction. Daily stress could compound sexual health difficulties in an already burdened population – couples with SIAD.

### Within-person associations between perceived stress and sexual health

Results at the within-person level were consistent with our hypotheses, as women and gender diverse individuals with SIAD’s greater daily perceived stress was related to their own and their partner’s lower sexual satisfaction and desire and higher sexual distress that same day. Likewise, partners’ greater daily perceived stress was linked to their own and the individual with SIAD’s lower sexual desire and sexual satisfaction, and their own (but not the individual with SIAD’s) higher sexual distress. That all pathways were significant with only one exception speaks to the robustness of these findings. Results are in line with interdependence theory stating that partners mutually influence each other’s outcomes (Kelley & Thibault, 1978). Our findings are also consistent with past cross-sectional and qualitative evidence among non-clinical samples (Bodenmann et al., 2010; Ferreira et al., 2015; Kalmbach et al., 2019; Mark & Lasslo, 2018), and extend that work by examining numerous indicators of sexual health simultaneously and using a dyadic and daily design in a sample of committed couples coping with SIAD.

One pathway to explain how perceived stress might lead to lower sexual health in both members of the dyad coping with SIAD is by decreasing the quality of their relationship interactions (Belu et al., 2023; Rancourt et al., 2017). Indeed, daily stress may amplify existing negative mood states surrounding an already sensitive topic – low sexual desire/arousal (Dubé et al., 2019; Jodouin et al., 2021). In turn, heightened unpleasant feelings could foster negative communication patterns in couples, including withdrawal and hostility (Randall & Bodenmann, 2009), resulting in more negative emotions and evaluations of the sexual relationship and lower interest in sex. Such patterns of sexual communication have been linked to poorer sexual outcomes in couples affected by painful intercourse, which is a sexual dysfunction that is often accompanied by low desire (Rancourt et al., 2017). Negative interpersonal interactions spurred by daily stress could heighten the perceived threat of a sexual problem such as guilt upon a partner’s initiation, or rejection if a sexual advance is turned down. Increased emotional sensitivity and reactivity could negatively affect how couples cope with SIAD and lead to poorer sexual health. These potential mechanisms (e.g., emotion regulation and communication patterns) should be investigated in future research.

### Between-person associations between perceived stress and sexual health

When measures were averaged across the 56 days, we found fewer effects overall relative to within-person. Still, similar patterns of associations between perceived stress and sexual satisfaction and distress emerged, while results for sexual desire were mixed. Specifically, individuals with SIAD who reported greater perceived stress on average reported higher sexual distress themselves (but not lower sexual satisfaction or desire) and their partners reported higher sexual desire (but not lower sexual satisfaction or higher distress). In addition, partners who reported greater perceived stress on average tended to report lower sexual satisfaction and higher sexual distress themselves (but not lower sexual desire) and the individuals with SIAD reported higher sexual distress (but not lower sexual satisfaction or desire). On average across the diary period, we observed the most consistent patterns for sexual distress, which is key to a SIAD diagnosis. In addition, partners of those with SIAD tend to be more distressed sexually compared to partners of those without sexual dysfunction (Rosen et al., 2019) and couples’ discrepant sexual desire is linked to greater sexual distress both daily and over one year (Jodouin et al., 2021). We also noted an unexpected finding: the heightened perceived stress of individuals with SIAD was linked to their partner’s higher sexual desire. This result is consistent with mixed evidence in the literature concerning the association between stress and sexual desire and requires further replication (Mües & Nater, 2020). It is possible that the partner may see sexuality as a coping mechanism to promote intimacy with the individual with SIAD and potentially reduce their stress (Taylor, 2006). As the results are correlational, it is also possible that partners’ higher desire is in general experienced as a significant stressor for the individual with SIAD, heightening their overall perceived stress (Rosenkrantz & Mark, 2018).

Lastly, the difference across our within- and between-person results may reflect that the former is better suited to capture variations in fluctuating states such as stress, sexual satisfaction, desire, and distress (Mark, 2014; Rosen et al., 2018). Indeed, such variables can be influenced on the timescale of days based on contextual factors and personal life events. Our findings and interpretations echo the recent paradigm shift to within- rather than between-person methods to study variations in affect or states (Brose et al., 2015) and speaks to the fact that comparing participants’ levels to their own reports may yield more precise effects than comparing them to other individuals.

### Limitations, contributions, and future studies

Findings of this study should be considered in light of its limitations. First, our daily findings are correlational, which limits our ability to make causal interpretations. While controlling for the previous day’s outcomes allowed initial insight into directionality, future studies should investigate the bidirectional associations between stress and sexual health. Although the daily methodology reduced recall biases and enhanced ecological validity (Rosen et al., 2018), data were still self-reported. Also, we measured global perceived stress; future studies could examine the relative contributions of different types of stressors in couples’ lives (e.g., sexual, relational, financial, etc.) to provide deeper insights and help refine intervention strategies. Further, as stress requires a multidimensional assessment, experimental studies including specific biological stress markers might offer different insight into the associations between stress and sexual health (Ditzen et al., 2019). Future studies could also account for developmental-contextual aspects that can alter how partners shape each other's relationships and health during stressful events, such as age, gender, culture, race/ethnicity, adversity, disability, or socioeconomic status (Shrout, 2021).

Nevertheless, this study has important theoretical and clinical implications as it highlights the interdependence between romantic partners’ stress and sexual outcomes in the context of SIAD. Findings support the Interpersonal Emotion Regulation Model of Women’s Sexual Dysfunction (Rosen & Bergeron, 2019) as they show the contribution of a proximal factor, daily stress, to sexual challenges and underscore the dyadic nature of these associations.

## Clinical implications and conclusions

The current findings demonstrate that in couples coping with SIAD, heightened perceived stress in one partner is linked to both partners’ sexual health outcomes. From a public health perspective, increasing couples’ ability to cope with stressors may be easier and more cost-effective than treating a cascade of individuals’ psychological or physiological responses to stress (Cohen et al., 2019). In line with current evidence-based clinical approaches for sexual health problems (Brown et al., 2019), our results suggest that enhancing dyadic stress management via targeted interventions may benefit sexual satisfaction and sexual desire and lower sexual distress in couples coping with SIAD. Dyadic coping (i.e., when partners jointly respond to a stressful situation) is a core concept in couples’ therapy when targeting stress (Bodenmann, 2005). Based on current evidence, interventions aiming to increase awareness and foster responsiveness, self-disclosure, positive mutual coping and capitalization in the context of daily stressful events may represent a fruitful avenue that could mitigate the impact of stress on SIAD sexual health outcomes (Bergeron et al., 2024; Bouchard et al., 2024). Specifically, therapists could help clients identify the types of stressors in their lives that contribute to SIAD symptoms and sexual dissatisfaction, such as an inequitable division of labor (Harris et al., 2022) or relationship strain Shimizu et al., (2024). Therapy could focus on ways to approach these challenges in a constructive way via improved communication skills as well as problem-solving training (Bodenmann, 2010). Stress could also be targeted clinically in couples coping with SIAD through mindfulness-based strategies including mindful breathing or body scans (Brotto & Smith, 2021), which have been found beneficial for the treatment of SIAD (Brotto et al., 2021).”

## Declaration of competing interest

The authors have no conflicts of interest to declare.

## Appendix Supplementary materials

Image, application 1

## Footnotes

1 The DSM-5 diagnosis is *Female* Sexual Interest Arousal Disorder. Women who are not female (e.g., trans women, intersex) and non-binary individuals can also meet the diagnostic criteria and were included in this study. Consistent with feedback from our past participants, patient partners, and best practices (e.g., Brotto & Galea, 2022), we omit “female” from this label and refer to participants as women and gender diverse individuals with SIAD.
