# Supplementary material for: Daily perceived stress and sexual health in couples with sexual interest/arousal disorder
Source: Int J Clin Health Psychol. 2025 May 13;25(2):100582. doi: 10.1016/j.ijchp.2025.100582 (PMC12141049; doi:10.1016/j.ijchp.2025.100582)
Supplement: Supplementary file 1 [file mmc1.docx]

**Figure S1**

*Participants flowchart*

**Figure S2**

*Diagram of daily within-subjects associations between perceived stress and sexual health outcomes for SIAD individuals and partners*


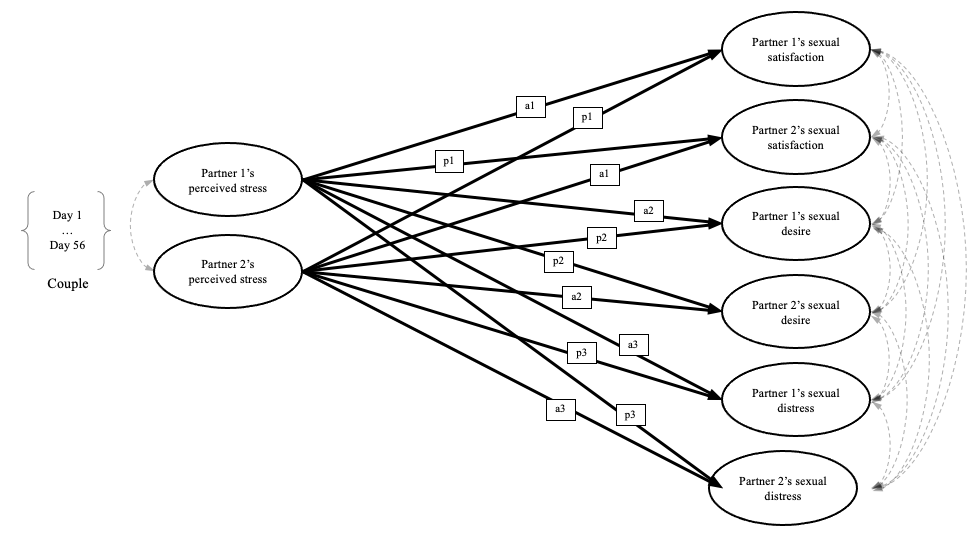


Note. On the within-person level, days were nested in couples such that there were 56 days of data for each variable, and time and yesterday’s outcomes were included in the model as covariates.

**Table S1**

*Between-person correlations between daily variables and descriptive statistics separately by partner (N = 229 couples)*

|  | 1 | 2 | 3 | 4 |
| --- | --- | --- | --- | --- |
| 1. Perceived Stress | **.35**** | -.14** | -.07** | .29** |
| 1. Sexual Satisfaction | -.18** | **.50**** | .39** | -.21** |
| 1. Sexual Desire | -.01 | .14** | **.19**** | .05** |
| 1. Sexual Distress | .41** | -.38** | .24** | **.28**** |
| Individuals with SIAD *M* (SD) | *1.56* (0.63) | *4.01* (1.06) | *1.70* (0.60) | *1.05* (0.84) |
| Partners *M* (SD) | *1.33* (0.65) | *4.10* (1.21) | *3.23* (1.13) | *0.71* (0.77) |
| Range | 0-4 | 1-7 | 1-7 | 0-4 |

*Note:* Between-person correlations reflect correlations between average scores across the diary period. Correlations for women and gender diverse individuals with SIAD are above the diagonal; correlations for partners are below the diagonal; bold correlations on the diagonal are between women and gender diverse individuals with SIAD and partners.

*** *p* < .001; ** *p* < .01; *p* < .05.
